# Laser-Induced Reconfiguration of Magnetic Domain Structure in Iron Garnet Films with Strong In-Plane Anisotropy

**DOI:** 10.3390/nano15231830

**Published:** 2025-12-04

**Authors:** Mikhail A. Stepanov, Nikolai V. Mitetelo, Andrey A. Guskov, Alexey S. Kaminskiy, Alexander P. Pyatakov

**Affiliations:** 1 Department of Nanoelectronics, Russian Technological University MIREA, Moscow 119454, Russia; 2 Faculty of Physics, M.V. Lomonosov Moscow State University, Moscow 119991, Russia

**Keywords:** laser-induced domain reconfiguration, bismuth-substituted iron-garnet, domain wall pinning and depinning, magnetic anisotropy

## Abstract

In this work we demonstrate the laser-driven reconfiguration of stripe domains in a thick bismuth-substituted iron garnet film with the (210) crystallographic orientation exhibiting strong in-plane anisotropy. Under a weak in-plane external magnetic field (H_‖_), laser irradiation leads to local “twisting” of the magnetic domains; domains with opposite magnetization rotate in different directions. The twisting angle increases linearly with the in-plane magnetic field (H_‖_) (above a threshold of approximately 6 Oe) and also changes linearly with the average laser intensity, being fully reversible after the irradiation process. The magnitude of the domain rotation effect does not depend on the light polarization state or its orientation. After optical irradiation, the magnetization distribution in the sample returns to its initial state. It is also observed that moving the focused beam spot along the surface can lead to irreversible modifications in the domain topology in several ways: there is a shift in the dislocations in stripe domain structure (domain “heads”) across the beam transfer direction, expanding the area with a specific magnetization vector orientation, and the stabilization of domain wall positions by their pinning on crystallographic defects. The proposed analytical model based on a local reducing of the effective anisotropy fully describes the rotation type and angle of domains and domain walls, defining their possible trajectories and certain values of the area heating or local anisotropy modulation and the rotation angles. The experimental results and the theoretical model demonstrate a thermal origin of the laser-induced effect in this type of magnetic domain structure.

## 1. Introduction

Precise control of the spatial distribution of magnetization in magnetic dielectrics, particularly in iron garnet films with stripe domain patterns, is a challenging task nowadays, especially in the research area focused on the creation of miniaturized, reconfigurable, and energy-efficient micromagnetic architectures for photonics and spintronic applications [1,2,3]. In this type of devices, domain walls act as functional elements, serving as magnonic waveguides, interfaces separating regions with distinct optical and magneto-optical response [4], and nanoscale-localized memory or logic elements [5,6]. In this context, iron garnet films are commonly used [7,8] due to their low coercivity [9], large magneto-optical response with high magnetic contrast, low optical losses [10,11] and pronounced magnetic anisotropy leading to magnetoelectric interaction [12,13].

Earlier investigations have shown that optical illumination of the domain structure in iron garnet films with a (111) crystallographic orientation and low uniaxial magnetic anisotropy leads to the nucleation and temporal evolution of magnetic inhomogeneities localized in the domain wall region [14]. In recent works, iron garnet films with (210) and (110) crystallographic orientations, which exhibit strong magnetic anisotropy, have attracted researchers’ attention. Interest in this type of magnetic media is large due to the prospect of ultrafast optical control of magnetic order [15,16]. Published studies have focused on magnetization dynamics and on the possibility of nucleation of new micromagnetic states. Another challenging task is to describe how optical radiation interacts with the domain structure of iron garnet films with a complex anisotropy state, since this effect enables precise displacement of regions with both uniform and inhomogeneous magnetization. In such films, optical excitation can be combined with magnetoelectric effects in order to achieve precise positioning of domains and domain walls [17,18].

Optical control of magnetic order can manifest itself via several physical mechanisms. The first is the inverse Faraday effect [19,20], in which circularly polarized light induces an effective magnetic field. Another mechanism is laser-induced heating [19,21], which locally decreases the magnetic anisotropy constant and modifies the micromagnetic configuration in external magnetic fields. In addition, photo-induced effects [22,23] associated with the generation of nonequilibrium charge carriers and local electric and magnetic fields can lead to local changes in the magnetic phase (for example, transitions and “paramagnetic-to-ferromagnetic” phase shift [24]). All these mechanisms, separately and in combination, can provide different approaches to new devices.

In this work, we investigate the optical manipulation of the micromagnetic structure of the iron garnet film. The main task of this research is to find a possibility and experimental conditions for precise control over the domain pattern and domain walls and to provide the analytical description with effect’s value calculations for defining mechanisms of light–matter interaction in a magnetic medium under intense laser irradiation.

## 2. Materials and Methods

**Figure 1:**
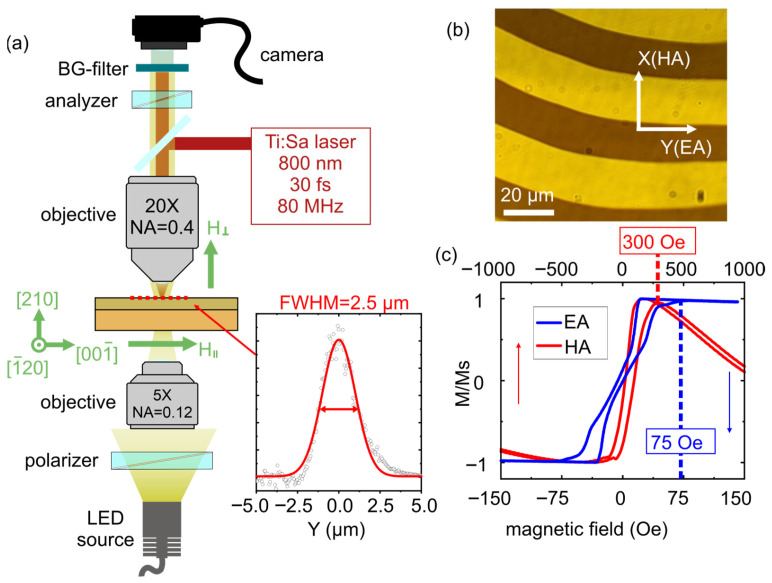
(**a**) Scheme of the transmission polarization microscopy experimental setup exhibiting the possibility of focusing the laser beam onto the sample’s surface with highlighted types of crystallographic orientations; the inset shows the beam profile at the focusing spot (measured by “knife-edge” method). (**b**) Image of the sample’s domain structure. (**c**) Hysteresis loops measured in the longitudinal magneto-optical Kerr effect (MOKE) geometry with the external field applied along the in-plane easy axis (EA) and along the hard axis (HA).

The film of (BiLu)_3_(FeGa)_5_O_12_ iron garnet with a thickness of about 10 µm, grown by liquid-phase epitaxy on a gadolinium gallium garnet (GGG) substrate, was used in the experiments. This film belongs to the crystallographic point group *m* and has a (210) crystallographic orientation (as was previously shown for this sample in work [12]). The micromagnetic structure was visualized using “white light” illumination and transmission polarization microscopy (Faraday geometry) (Figure 1a) with a WITec alpha 300S microscope (Ulm, Germany). Optical excitation was provided by a femtosecond titanium-sapphire (Ti:Sa) laser TiF-DP (Avesta; Troitsk, Moscow, Russia) with a wavelength of 800 nm operating at an 80 MHz repetition rate and a 30 fs pulse duration, as well as in the continuous-wave operation mode of the same laser system. Focusing on the free-standing film was performed with a Zeiss (Oberkochen, Germany) EC Epiplan HD objective (20×, NA = 0.4). To focus the white light source in the crossed polarizer–analyzer configuration, a Zeiss (Oberkochen, Germany) A-Plan objective (5×, NA = 0.12) was used. The laser beam diameter was approximately 5 µm (beam profile measured by the “knife-edge” method is shown in the inset in Figure 1a). Polarization of the laser beam was configured using a half-wave and a quarter-wave plate, enabling linearly polarized light at various orientations of the polarization plane, as well as right and left circular polarization states. An optical filter (SCHOTT (Mainz, Germany) BG39) was incorporated into the experimental setup to visualize effects associated with optical excitation of the structure in the focal region of the beam. The sample’s micromagnetic structure was modulated by external magnetic fields applied by electromagnets either perpendicular to the sample’s surface (H_⊥_, “out-of-plane”) or in the plane of the sample (H_‖_) perpendicular to the stripe domains.

When the external field is at a value of zero, the film has a stripe domain pattern with a period of approximately 40 µm (Figure 1b). The domains are aligned along the in-plane projection of the easy axis (EA). For films of such thickness, the domain configuration is known to be essentially uniform along the whole film thickness. The strong in-plane magnetic anisotropy is confirmed by the longitudinal magneto-optical Kerr effect (MOKE) hysteresis loops measured with the external field applied parallel to the domains (EA in Figure 1c) and perpendicular to it (hard axis (HA) in Figure 1c). The field values required to reconfigure the film into a single-domain state are about 75 Oe along the EA and 300 Oe along the HA (these values are highlighted in the hysteresis loops shown in Figure 1c).

## 3. Experiment

**Figure 2:**
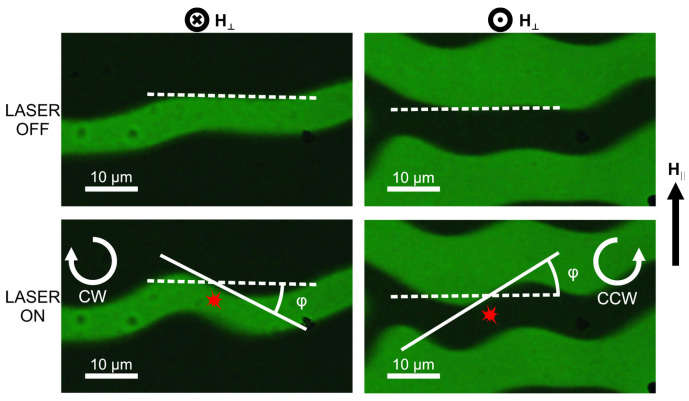
Illustration of laser-induced domain twisting. Left figures: *z*-component of the magnetization is oriented towards the sample’s substrate; right figures: *z*-component of the magnetization is oriented out of the sample. Top panels—the structure before optical irradiation; bottom panels—during illumination with a focused laser beam. Red point corresponds to the illumination spot, dotted and solid lines denote to initial and modified domain wall orientation, and arrows show the “twisting” direction of the magnetic domains.

During the experimental investigations, we detect the local response of the micromagnetic structure to laser irradiation. During laser irradiation of the domain center (marked with red in Figure 2), its azimuthal rotation is observed: the domain reorients in the clockwise (CW) or counterclockwise (CCW) direction depending on the magnetization orientation in a domain under illumination. The response reaches the largest rotation angle value when the focusing spot is at the domain center. The type of twisting is particularly determined by the initial magnetization at the excited sample’s region: for a fixed in-plane field H_‖_, a domain in which magnetization is oriented toward the film surface (+z) rotates in the CW direction, whereas a domain in which the magnetization vector is oriented toward the substrate (−z) rotates in the CCW direction. Consequently, 180° domains exhibit opposite signs of the twisting angle φ, as indicated in Figure 2, and this value is defined by calculating the mutual orientation of white straight lines drawn along the domain wall before and after irradiation (Figure 2). It is important to note that the out-of-plane magnetic field H_⊥_ is used to stabilize the domain size and equalize the domain period in the laser-irradiated region and to provide identical experimental conditions. The external in-plane field is varied to control the magnitude of the rotation angle. The out-of-plane field component (H_⊥_) does not affect the sign or magnitude of the domain twisting angle. After optical exposure, the micromagnetic texture returns to its initial state and the effect is reproducible during several repeated irradiation cycles. Notably, the effect has the same magnitude under continuous-wave and femtosecond regimes of laser operation, which proves a thermal origin of the observed response. The observed effect is quantitatively and qualitatively reproducible at all sample areas with a stripe domain structure without crystallographic defects.

**Figure 3:**
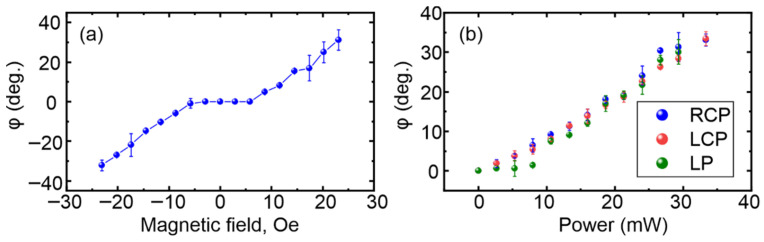
(**a**) Stripe domain rotation angle dependence on the magnitude of the in-plane external magnetic field (optical power in this experiment was 30 mW); (**b**) twisting angle dependence on the optical power for right-handed (RCP), left-handed (LCP) circular, and linear (LP) laser polarizations (in-plane magnetic field magnitude in this experiment was 20 Oe).

The observed effect is characterized by the rotation angle φ of the domain wall under optical illumination. The calculation for the rotation angle is performed using image processing: at first the initial position of the domain wall is determined and approximated by a straight line; after this calculation, the position of the twisted domain wall in the image obtained after optical irradiation is performed by a similar technique, and the angle between these two lines defines the twisting angle. For the determination of the numerical value of this angle (related to Figure 3), averaging is performed over several measurements. Figure 2 schematically illustrates the method for determination of this value. To establish the origin of the irradiation-induced modification of the micromagnetic structure, we measure the dependence of the domain rotation angle on the in-plane external magnetic field (H_‖_). The average optical power is about 30 mW, corresponding to an energy density of approximately 4.5 kJ/cm^2^. In the range of |H_‖_| ≤ 6 Oe, the response of the magnetic structure remains below the detection threshold, being undistinguishable. With increasing |H_‖_|, the rotation angle enlarges linearly with the field magnitude, and the sign of the in-plain H_‖_ establishes the direction of rotation (the sign of the angle φ). We also determine the dependence of the rotation angle on the optical power and the polarization state of the beam (right- and left-handed circular (RCP/LCP) and linear (LP)) in an external magnetic field of about 20 Oe. As shown in Figure 3b, in the investigated laser power range the response increases linearly with light intensity, and it is also evident that no dependence on the polarization of the irradiation is observed. Notably, the value of the effect does not depend on the orientation of the linear polarization plane or its helicity which can lead to an inverse Faraday effect of local field generation. It means that during heating, the magnetic domain structure is not sensitive to the magnetic field of the electromagnetic wave or to the weak helicity-induced “Faraday field” [19]. It is also worth noting that maximal optical power used in the experiment (34 mW) does not lead to any structural or morphological changes in the sample, which was obtained by optical polarization microscopy.

## 4. Theoretical Description and Calculation

To establish the origin of the effect, it should be noted that the investigated films had an orthorhombic crystal lattice exhibiting strong anisotropy, which determined the easy and hard axes in the film plane and the orientation of the stripe domain structure. Additionally, it is necessary to mention that the presence of the in-plane magnetic field perpendicular to the domain boundaries should be taken into account.

**Figure 4:**
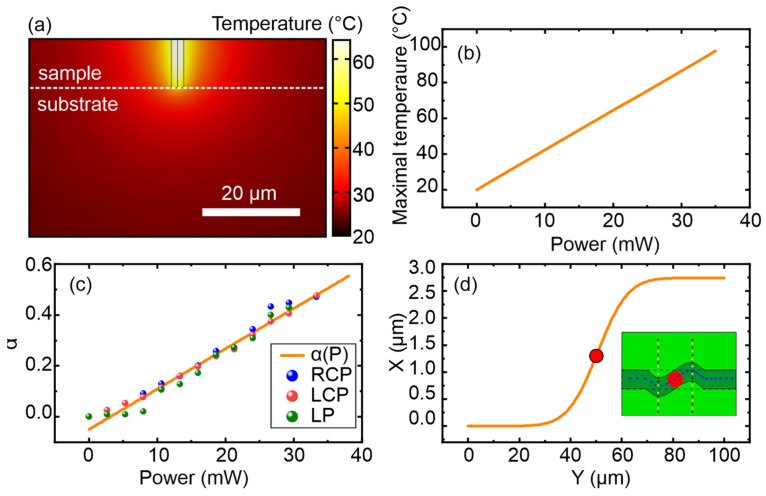
(**a**) Temperature distribution in the plane perpendicular to the sample surface at a laser power of 20 mW. (**b**) Dependence of the maximal heating temperature on the laser power. (**c**) Dependence of the relative reduced anisotropy constant on laser power (with an in-plane field magnitude of 20 Oe). (**d**) Calculated domain wall trajectory at a laser power of 3 mW under an in-plane external magnetic field of 20 Oe (red point shows the irradiation spot, blue arrows illustrate “in plane magnetization vector direction).

The heating temperature in the laser area was estimated by numerical simulation using the COMSOL Multiphysics (5.2 version) software. In the simulation, the heating by the laser beam was replaced by a distributed heat source with the size of the laser beam, and the heating power was calculated analytically using the Bouguer–Lambert–Beer law taking into account the typical absorption for garnets [25]. Figure 4a shows the calculated temperature distribution in the plane of the sample perpendicular to the surface at a laser power of 20 mW. As shown in the figure, the largest temperature in the heating area reached approximately 60 °C. The heating area had a characteristic size of about 10 µm, which is much smaller than the size of the observed domain structures. Also, as a result of the simulation, the dependence of the maximum heating temperature on the laser power was obtained and is shown in Figure 4b.

The dependence of the saturation magnetization on temperature in garnets has already been studied in previous papers; in [26], it is shown that in this temperature range, the change in the saturation magnetization of yttrium iron garnet does not exceed 20% of its value. The saturation magnetization only contributes to the magnetostatic energy, which is smaller compared to other terms (anisotropy, exchange) that depend only on the direction of the magnetic moment, since the heating area is small. In addition, since the domain structure of the sample is observed using the magneto-optical Faraday effect, a significant change in the saturation magnetization caused by heating would manifest as a change in the brightness of the heating area in the image. However, this is not observed, which allows us to neglect the change in saturation magnetization when explaining the effects described in the paper.

The change in the magnetic anisotropy constants has also been studied in the literature; for example, in bismuth-substituted iron garnets in the temperature range reached in the experiment, the anisotropy constants decreased almost linearly with increasing temperature [27].

The origin of the effect can be described as follows: heating induced by tightly focused laser radiation leads to a local reduction in the orthorhombic anisotropy; the direction corresponding to the minimum of the domain wall energy becomes no longer fixed to the orthorhombic (−120) axis, and the external magnetic field reconfigures the magnetization vector in the domain and its domain walls, resulting in azimuthal rotation of the magnetic domain structure in the area close to the illumination region. The rotation angle φ observed in the experiment in this case corresponds to the angle between the in-plane easy axis of the investigated sample and the magnetization vector orientation under the beam.

To explain the different directions of azimuthal rotation in domains with antiparallel magnetization directions, it should be considered that in films with orthorhombic anisotropy, magnetization has a significant in-plane magnetic vector component (in this sample it is almost equal to the normal component), and its direction in two adjacent domains is opposite. Therefore, the external magnetic field exhibits opposite effects on neighboring domains.

To determine the rotation angle of magnetic domains in the structure, the energy of a stripe domain should be written as an integral over the magnetic domain length L:

(1) $$W=\int_{0}^{L}\left(E_{anis}+E_{H}+E_{DW}\right)dl,$$

where three terms correspond to the anisotropy energy ($E_{anis}$), energy in applied magnetic field **H** ($E_{H}$), and the domain wall energy ($E_{DW}$).

The first term can be expressed in terms of the effective anisotropy constant. ($K_{eff})$:

(2) $$E_{anis}=-K_{eff}hd{{\left(\sin\theta_{0}\right)}^{2}\left(\sin\varphi \left(\vec{r}\right)\right)}^{2},$$

where *h* = 10 μm (film thickness); d = 20 μm—domain width; and φ—the angle between the in-plane component of the magnetization and the component of EA in the sample’s plane (*y*-axis in Figure 1b). The effective anisotropy can be represented as the sum of two contributions: orthorhombic and uniaxial, while their magnitudes can be estimated as 3 × 10^−4^ J/cm^3^ [12,28]. Angle $\theta_{0}$ defines the direction of the magnetization in the domains relative to the sample’s surface perpendicular.

In the external magnetic field applied along the *x*-axis, the in-plane component of the domain magnetization tends to reorient and the domain walls also reconfigure to minimize the magnetostatic energy of the wall ($E_{DW}$), while the *x*-component of the domain’s magnetization remains zero. Thus, in addition to the anisotropy contribution, the domain energy per length unit possesses the Zeeman term and the domain wall energy.

(3) $$E_{H}=-M_{S_{∥}}Hhd\cos\varphi (\;\vec{r});$$

(4) $$E_{DW}=4\sqrt{K_{eff}(\vec{r})A}\cdot h$$

where $M_{S_{∥}}$ is saturation magnetization (in-plane component) ${(M}_{S_{∥}}=$ 640 Oe, $A=3.3\cdot 10^{-14}J/cm$—exchange stiffness constant, *h*—film thickness, and d= 20 μm—domain width).

The effective anisotropy constant is a function of position: it reaches its minimum at the beam focusing spot center and has a Gaussian profile with a corresponding radius of about 2.5 μm from the center.

(5) $$K_{eff}\left(\vec{r}\right)=\left(1-\alpha \left(P\right)\cdot exp\left(-\frac{{\left|\vec{r}-\vec{R_{0}}\right|}^{2}}{2r_{0}^{2}}\right)\right)\cdot K_{0}$$

where $\vec{R_{0}}$—radius vector directed to the spot, $r_{0}=2.5$ μm—radius of the focused laser beam spot, and $\alpha \left(P\right)$—the relative reduction factor of the anisotropy at the beam center which depends on the laser power.

Minimization of the energy-functional W based on Equations (1)–(5) for random units of the coefficient α makes it possible to determine the calculated dependence φ( α) of the twisting angle of the magnetization in the domain at the beam center with respect to the *y*-axis after the corresponding experimental dependence $\varphi \left(P\right)$ has already been measured. The desired dependence $\alpha \left(P\right)$, shown in Figure 4c, can thus be calculated. Figure 4c also presents values recalculated from the experimental data revealing that the in-plane anisotropy at the beam focusing spot is reduced by approximately two times of its magnitude, which fully corresponds to the experimentally measured domain rotation angle of 30° in an external magnetic field of about 20 Oe. The obtained dependence offers the possibility to determine the domain wall trajectory for a specified power value by minimization of the energy-functional W. Figure 4d shows the calculated domain wall trajectory at an optical power of approximately 3 mW in an external field of about 20 Oe, and the inset illustrates the corresponding domain configuration (red dots show laser beam focusing spot; blue arrows illustrate “in plane” magnetization vector direction). The proposed analytical model based on a local reducing of the effective anisotropy fully describes the rotation type and angle of domains and domain walls and also defines their possible trajectory types, certain values of the area heat, or local anisotropy modulation and rotation angle qualities. This result indicates that the origin of the interaction of optical radiation with the magnetic system is dominantly thermal.

## 5. Other Methods of Magnetic Domain Structure Reconfiguration

**Figure 5:**
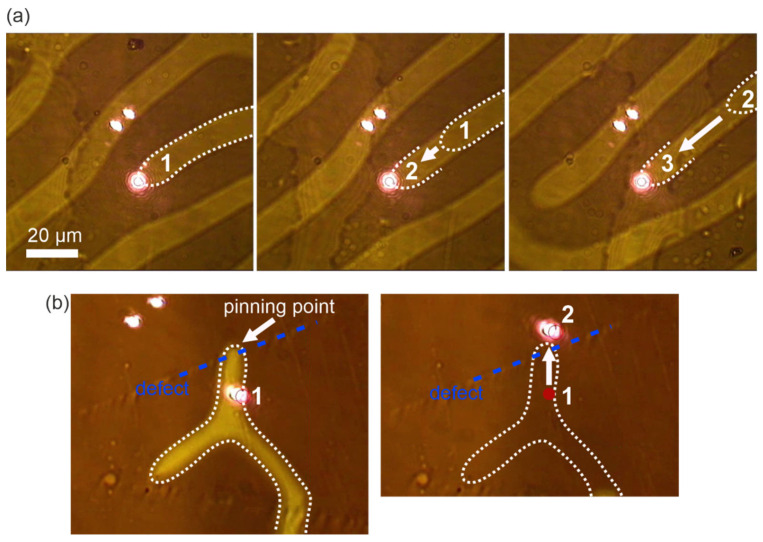
(**a**) Visualization of a precisely tunable displacement of a domain head during beam scanning across the structure; (**b**) visualization of the response of a magnetic domain pinned near a crystallographic defect, demonstrating its laser-induced depinning and erasure (red point illustrates initial laser spot, blue dotted line denotes to the crystallographic defect position).

The demonstrated thermal mechanism of interaction between laser irradiation and the magnetic structure does not only induce the rotation of domains but also provides precise control and reconfiguration of the domain pattern. These effects can be observed when the focused laser beam is translated across/along the domain structure and the region of locally reduced anisotropy is translated with the beam, producing a permanent and stable modification of the local magnetic order if the illumination is switched off. The response is particularly pronounced at the dislocations of the stripe domain structure (domain “heads”). Figure 5a shows the displacement of a domain “head” (its positions 1 → 2 → 3 are highlighted by a white dashed line) following the trajectory of the beam translation (the displacement of the domain head is about 65 µm in the “out-of-plane” magnetic field of ~30 Oe). Notably, the domain “head” can be relocated in both directions along the stripe direction, enabling either a reduction or an expansion in the region with a certain magnetization vector orientation. After laser excitation is switched off, the domain walls remain near the final beam spot position and do not return to their initial state, indicating stabilization of the micromagnetic configuration after illumination. The similarity to the “rotation” effect is also evident: the displacement does not depend on the type of light polarization and the orientation of the out-of-plane external magnetic field (Figure 5a). This effect can be observed without an in-plane external field.

It is also possible to manipulate micromagnetic textures pinned (localized) at crystallographic defects. Figure 5b illustrates that tight focusing of the laser beam (moving from *point 1* to *point 2*) on a domain wall (boundary marked with white dashed line) pinned at a mechanical scratch on the crystal surface (blue dashed line along the defect visualized by optical polarization microscopy) leads to its erasure. This effect occurs because the applied out-of-plane magnetic field (about 80 Oe) has already saturated all domains, which are not pinned in the area near crystallographic defects, and optical illumination provides sufficient thermal assistance to overcome the pinning potential (so-called “depinning” [29]) leading to a pinned magnetic domain disappearance.

The effect of domain “head” displacement and domain wall depinning under laser irradiation has been demonstrated, but its quantitative characterization in terms of the wall shift per laser power and per beam transfer distance cannot be measured using common optical microscopy experimental conditions. So, the observed wall displacement induced by scanning of the focused laser beam can be consistently and qualitatively described with the help of a theoretical approach. When the laser beam passes through a domain wall, its surface energy in the beam area changes due to the decrease in anisotropy constants caused by heating, which makes the wall’s replacement occur in the direction of beam transfer. The displacement process itself can be qualitatively described by the following model. Let us consider a single Bloch-type domain wall located along the x-axis. Its surface energy density will be:

(6) $$\sigma_{w}=4\sqrt{AK_{eff}},$$

where A is the exchange energy constant and $K_{eff}$ is the effective magnetic anisotropy constant before heating.

Then the total energy of the domain wall in a film of thickness h in the area of a laser spot of size L before heating will be:

(7) $$E_{w1}=h\int \;\sigma_{w}(x)dx=4h\sqrt{AK_{eff}}L$$

During local heating of this area, the effective anisotropy constant will decrease by a value of $\Delta K_{eff}$, which will reduce the domain wall energy. As the spot moves away from the domain wall, the magnetization region denoting to a domain wall will begin to follow the laser beam because then its total energy will be lower (even with a larger value of domain wall length). At a certain critical increase in the wall length $\Delta L_{cr}$, its energy will be equal to the initial value:

(8) $$E_{w2}=4h\sqrt{A(K_{eff}-\Delta K_{eff})}(L+\Delta L_{cr})=E_{w1}$$

After this, further displacement of the domain wall will become unfavorable. Thus, from (7) and (8), one can estimate the critical increase in the wall length:

(9) $$\Delta L_{cr}=L\left(\sqrt{\frac{K_{eff}}{K_{eff}-\Delta K_{eff}}}-1\right)$$

or since $\Delta K_{eff}$ = $\alpha K_{eff}$ and α∼ P, as was already described in previous power dependent anisotropy reduction studies (Figure 4c), then:

(10) $$\Delta L_{cr}=L\left(\frac{1}{\sqrt{1-\alpha }}-1\right)∼L\left(\frac{1}{\sqrt{1-\beta P}}-1\right)$$

where β is the proportionality coefficient between the change in the effective anisotropy constant and incident laser power.

**Figure 6:**
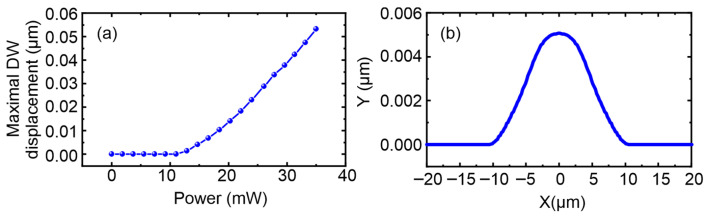
(**a**) Dependence of the largest domain wall (DW) displacement on laser power calculated from the theoretical model; (**b**) typical shape of the wall during its displacement (at a distance of 5 µm between the beam center and the initial DW position).

As the spot moves further away from the wall, the heating in the wall area will disappear, causing it to return to its initial position. Figure 6a shows the dependence of the largest wall displacement on the laser power, calculated for a Gaussian beam taking into account the obtained dependence $\alpha \left(P\right)$, while Figure 6b shows a typical wall shape during its displacement at a distance of 5 µm between the beam center and the initial wall position, showing the significant tendency of a domain wall to perform a transfer of a laser beam’s relocation direction. So, using this approach, we can perform a full analytical description of a domain wall transfer for certain incident power and reconstruct its shape during replacement. If on its “spontaneous” way back without laser irradiation the domain wall encounters a crystallographic defect that creates an energy barrier for it, pinning of the wall to this defect will occur and it will be a new localization position for the magnetic domain.

## 6. Conclusions

This work demonstrates precise laser-driven manipulation of magnetic order in a (210)-oriented bismuth-substituted iron garnet film by localized thermal decrease in magnetic anisotropy in the illuminated region. Such localized irradiation induces twisting-type reconfiguration of magnetic stripe domains and domain walls in weak magnetic fields. This effect increases linearly with the lateral external in-plane magnetic field above a certain threshold defined by the structure’s magnetic properties, changes linearly with the laser power, and is not sensitive to light polarization. Controllable and precise reorientation of the domain configuration is demonstrated by azimuthal rotation of domains and directed displacement of domain walls in the area of local irradiation. The role of crystallographic defects in obtaining the required stable domain configuration is analytically described and visualized. These effects are promising for photonics and spintronics, enabling control of magneto-optical response and magnetic dynamics for spin–charge current conversion during terahertz emission and also for magnetoelectric and flexomagnetoelectric effects used for the creation of controllable logic and memory devices.

## Data Availability

The original contributions presented in this study are included in the article. Further inquiries can be directed to the corresponding author.
